# Resection of hepaticocystic duct which is a rare anomaly of the extrahepatic biliary system: a case report

**DOI:** 10.1186/1752-1947-7-279

**Published:** 2013-12-30

**Authors:** Nobuhiro Harada, Yasuhiko Sugawara, Takeaki Ishizawa, Junichi Kaneko, Yoshihiro Sakamoto, Taku Aoki, Kiyoshi Hasegawa, Norihiro Kokudo

**Affiliations:** 1 Hepatobiliary Pancreatic Surgery Division and Artificial Organ and Transplantation Division, Department of Surgery, Graduate School of Medicine, University of Tokyo, 7-3-1 Hongo, Bunkyo-ku, Tokyo 113-8655, Japan

**Keywords:** Abnormalities of the cystic duct, Anomaly, Biliary system, Cholecystectomy, Cholecystohepatic, Hepaticocystic

## Abstract

**Introduction:**

There are several variations in the morphologic characteristics of the extrahepatic biliary system. A hepaticocystic duct is one of the rare variations.

**Case presentation:**

A 69-year-old Asian man underwent a cholecystectomy for cholelithiasis. His cystic duct was not detected during surgery. An intraoperative cholangiography revealed that his common hepatic ducts drained directly into the neck of his gallbladder. There was no common bile duct, as evidenced by the union of the common hepatic and cystic ducts.

**Conclusion:**

Knowledge of anomalies related to the extrahepatic biliary system is important for decreasing the severe morbidity and mortality that may result from a failure to recognize the anomaly.

## Introduction

There are various anomalies of the extrahepatic biliary tree. In 1882, the surgical anatomy of the area was investigated with the advent of cholecystectomy. The lack of awareness of such anomalies during surgery of the biliary system may result in iatrogenic injuries. Here we describe a case in which a patient with a hepaticocystic duct underwent cholecystectomy.

## Case presentation

A 69-year-old Asian man was admitted to our hospital for complaints of epigastric pain. There had been no nausea, vomiting, or melena. At the age of 67, he had a femoral head replacement for aseptic necrosis of his femoral head, and had been treated with oral medicine for gout since the age of 65. A physical examination revealed no abnormal signs other than jaundice and mild fever.

**Figure 1:**
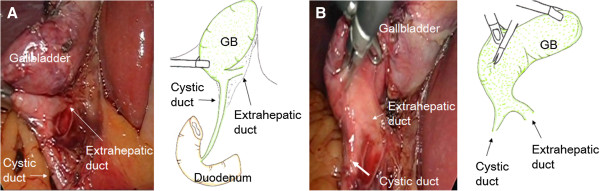
**Intraoperative laparoscopic photographs and illustrations.** The anatomic relationship between all structures in Calot’s triangle could be easily identified. Abbreviation: GB, gallbladder.

Laboratory data revealed elevated serum hepatobiliary enzyme levels (alanine aminotransferase 247IU/L, aspartate transaminase 123IU/L, γ-guanosine triphosphate 803IU/L, total bilirubin 2.3mg/dL, direct bilirubin 1.2mg/dL), a white blood cell count of 10,200/μL, and serum C-reactive protein levels of 0.24mg/dL. Ultrasonography revealed dilatation of his intrahepatic bile duct and gallstones. Computed tomography revealed stones in his common bile duct. The diameter of the largest stone was 15mm. The wall of his gall bladder was not thickened (Figure 1).

His common bile duct stones were removed by endoscopic retrograde cholangiopancreatography (ERCP) before surgery. ERCP revealed that the diameter of his common bile duct was 11mm and the size of the filling defect in his inferior common bile duct was 15 × 10mm. His intrahepatic bile duct was dilated because of the stones in his common bile duct and cholangitis developed. The anatomy of his biliary tree was not closely investigated in the ERCP.

**Figure 2:**
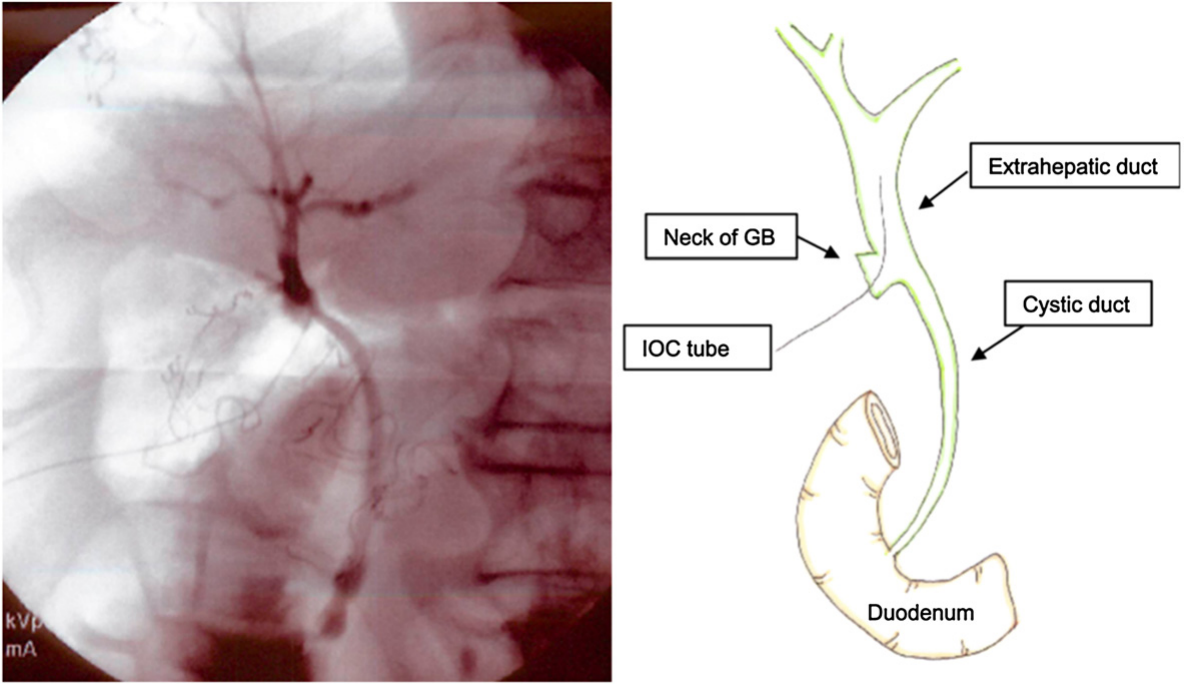
**Intraoperative cholangiography image which was taken after cholecystectomy.** The tube was placed from the neck of the gallbladder into the extrahepatic duct. Abbreviation: GB, gallbladder; IOC, intraoperative cholangiography.

Cholecystectomy was first attempted laparoscopically. The anatomic relationships between all structures in Calot’s triangle were easily identified (Figure 1). His cystic duct, however, could not be found during surgery. Therefore, the surgery was converted to an open surgery. We mobilized his gallbladder from his liver, at first. We secondly performed a partial cholecystectomy at the body of his gallbladder getting enough distance from his hepatic duct. The neck of his gallbladder was closed by interrupted suture using 4–0 Vicryl after two stones were removed (diameter: 10mm and 15mm, respectively). Intraoperative cholangiography (Figure 2) did not reveal a cystic duct; he was diagnosed with a hepaticocystic duct type IIIB [1]. There were no findings suggesting Mirizzi syndrome.

A histologic study of his resected gallbladder indicated mild inflammatory changes without malignancy, compatible with cholecystitis. He was discharged from our hospital on postoperative day 10 without complications. Two months after the surgery, however, he was readmitted to the hospital for fever and jaundice due to bile leakage from the closing point of his gallbladder. He was diagnosed with obstructive jaundice due to a stone remaining in the cystic duct, which was removed by ERCP. He has now been followed up for 1.5 years without biliary complications.

## Discussion

Walton [2] reported the first case with cystic duct abnormalities. In 1958, Braasch [3] categorized all biliary tract anomalies reported so far with cholecystohepatic ducts being categorized as the rarest type. The incidence of hepaticocystic ducts is not clear, although the incidence of congenital anomalies of the extrahepatic biliary system in general is reported to be from 0.58%- to 47.2% [4]. Knowledge of embryology of the biliary tract is necessary to understand the hepaticocystic duct. The hepatic diverticulum arises from an area on the ventral aspect of the gut, at the junction between the foregut and the midgut. The hepatic diverticulum is divided into two parts; a cranial part rise to the liver and the hepatic and common hepatic ducts, and a caudal part rise to the gallbladder and cystic duct. The common bile duct is derived from the antrum which is the common portion of the hepatic diverticulum [5]. Losanoff *et al.*[6] postulated that this anomaly was due to failed cell proliferation from the common bile duct and advocated naming this anomalous extrahepatic biliary system a ‘hepaticocystic duct’. Due to this anomaly, the gallbladder is continuous with the proximal part of the extrahepatic biliary system, and thus the cystic duct is not carried away from the duodenum and the common bile duct remains undeveloped.

A hepaticocystic duct drains both the left and right hepatic ducts into the gallbladder, with agenesis of the common bile duct, and the cystic duct drains the entire biliary system into the duodenum [1,7]. Therefore, the hepaticocystic duct must be differentiated from the accessory ducts. Lamah *et al.*[8] described that a hepaticocystic duct draining both hepatic ducts into the gallbladder neck should be included in the category of an accessary bile duct as a hepatic duct anomaly.

There are three reports [9-11] of biliary operations in patients with a hepaticocystic duct. The indications for surgery were gallstones and cholecystitis. In two cases, the selected surgical procedure was a partial cholecystectomy [9,10]. In the other case, the biliary tree was reconstructed using a hepaticojejunostomy (Roux-en-Y loop) [11]. Williams and Williams [12] reported the surgical procedure and outcome for gallstones and cholecystitis with abnormalities of the bile ducts in four cases. Of these, a hepaticocystic duct was found in one patient. The surgeons resected the anomalous cystic duct, but could not determine whether the duct acted as a bile elimination tract during or after the operation. When an abnormality such as observed in this case is not detected during surgery and the cystic duct is resected, biliary reconstruction using a hepaticojejunostomy (Roux-en-Y loop) is necessary to ensure elimination of the bile. However, if an anomaly is detected before resection of the cystic duct, a partial cholecystectomy and choledochoplasty may be considered the procedure of choice.

## Conclusions

A hepaticocystic duct is a rare anomaly. Knowledge of anomalies related to the extrahepatic biliary system during surgery is important for decreasing the severe morbidity and mortality that may result from a failure to recognize an anomaly.

## Consent

Written informed consent was obtained from the patient for publication of this case report and accompanying images. A copy of the written consent is available for review by the Editor-in-Chief of this journal.

## Competing interests

The authors declare that they have no competing interests.

## Authors’ contribution

NH, YSu and TI analyzed and interpreted the patient data. YSa, JK, TA, KH and NK contributed to writing the manuscript. All authors read and approved the final manuscript.
